# Effects of Lactic Acid Bacteria Fermentation on the Physicochemical Properties of Rice Flour and Rice Starch and on the Anti-Staling of Rice Bread

**DOI:** 10.3390/foods12203818

**Published:** 2023-10-18

**Authors:** Xinlai Dou, Xuyang Ren, Qiumei Zheng, Yinyuan He, Mingshou Lv, Linlin Liu, Ping Yang, Yanlin Hao, Fenglian Chen, Xiaozhi Tang

**Affiliations:** 1 College of Food Engineering, Harbin University of Commerce, Harbin 150076, China; hsddxl2024@163.com (X.D.); rxyhsd@163.com (X.R.); nkddxl@163.com (Q.Z.); heyinyuan717@163.com (Y.H.); lumingshou@hrbcu.edu.cn (M.L.); keaiduolinlin@126.com (L.L.); ping8434@163.com (P.Y.); 2 Institute of Nutrition and Health, China Agricultural Universities, Beijing 100083, China; haoyl@cau.edu.cn; 3 College of Food Science and Engineering/Collaborative Innovation Center for Modern Grain Circulation and Safety/Key Laboratory of Grains and Oils Quality Control and Processing, Nanjing University of Finance and Economics, Nanjing 210023, China

**Keywords:** lactic acid bacteria fermentation, rice bread, anti-staling, starch, japonica rice

## Abstract

In this study, *Lactococcus lactis lactis* subspecies 1.2472, *Streptococcus thermophilus* 1.2718, and thermostable *Lactobacillus rhamnosus* HCUL 1.1901–1912 were used to ferment rice flour for preparing rice bread. The characteristics of fermented rice bread were studied to elucidate the mechanism by which fermentation improves the anti-staling ability of rice bread. The amylose content of rice flour increased after fermentation. The peak viscosity, attenuation value, final viscosity, recovery value, and gelatinization temperature decreased. Amylopectin was partially hydrolyzed, and the amylose content decreased. The crystallinity of starch decreased, and the minimum crystallinity of *Lactococcus lactis* subsp. *lactis* fermented rice starch (LRS) was 11.64%. The thermal characteristics of fermented rice starch, including To, Tp, Tc, and ΔH, were lower than RS (rice starch), and the △H of LRS was the lowest. Meanwhile, LRS exhibited the best anti-staling ability, and with a staling degree of 43.22%. The T_22_ of the LRF rice flour dough was lower, and its moisture fluidity was the weakest, indicating that moisture was more closely combined with other components. The texture characteristics of fermented rice bread were improved; among these, LRF was the best: the hardness change value was 1.421 times, the elasticity decrease was 2.35%, and the chewability change was 47.07%. There, it provides a theoretical basis for improving the shelf life of bread.

## 1. Introduction

Rice is the most widely consumed cereal in the world, with nearly 4 billion people considering it as a staple food, accounting for 23% of their dietary energy intake [1]. Rice is a staple crop in China, boasting a wealth of nutritional elements that fulfill the body’s dietary requirements [2]. The nutritional value and gastronomic quality of rice dictate its reflected in the overall grain product landscape [3]. Primarily consumed as a staple food, rice is also processed to yield an array of convenient and expedient snack products with diverse flavors and styles [4]. Given that starch constitutes approximately 70% of the composition of rice, the staling phenomenon occurs unavoidably during processes such as transportation, storage, and sale, causing alterations in its intrinsic attributes, including decreased solubility in water, diminished palatability, and impaired digestive properties [5]. Freshly baked bread exhibits a desirable texture, is soft yet resilient in structure, and is elastic [6]. However, over the course of storage, bread experiences staling effects, transitioning from soft to firm and undergoing degradation in structural integrity, texture coarseness, diminished palatability, and loss of distinctive flavors [7,8].

The processing technology and composition of starch-based foods are significant factors that influence the staling of such products [9,10]. Currently, domestic research primarily focuses on altering storage conditions, selecting raw materials, and incorporating emulsifiers and enzyme preparations to inhibit the staling of starch-based foods [11,12]. Ding et al. [13] proposed that starches exhibit higher water content and staling occurs faster at a storage temperature of 4 °C and a water content of 60%. Qiu et al. [14] discovered that enzymatic treatment of rice bran starch resulted in a relative decrease in crystalline melting enthalpy and crystallinity, leading to the suppression of staling effects. The effectiveness of this process was influenced by the extent of enzymatic hydrolysis, and a certain degree of enzymatic hydrolysis could impede the staling of instant rice flour.

In recent years, numerous researchers worldwide have explored the anti-staling mechanisms of rice-based foods [15,16,17]. Many studies have observed that fermentation plays a certain role in the anti-staling properties of starch. Not only could it enhance the texture and aroma of rice bread and increase its fluffiness, but it could also extend its shelf life [18]. Tu et al. [19] reported that fermentation led to the surface erosion of starch granules, resulting in a denser structure, layered orderliness, relative crystallinity reduction, decreased double helix, short-range ordered structure, and reduced Mw (molecular weight). Cizeikiene et al. [20] showed that the application of thermophilic acid dough increased the porosity, elasticity, brittleness, and moisture content of bread while inhibiting fungal corruption on the surface of bread skin to improve the shelf life of bread. Unfortunately, research on the anti-staling effects of fermented rice bread is limited. Although there have been numerous studies on bread staling, research concerning rice flour bread fermented by lactic acid bacteria is relatively scarce, and bread fermented using *Lactococcus lactis* subsp. *lactis* and *Streptococcus thermophilus* is not reported yet. Therefore, this study investigated the anti-staling mechanisms of three types of lactic acid bacteria-fermented rice flour and rice starch and concurrently produced bread from them to examine the anti-staling capacity of bread. The aim of this study was to provide a theoretical foundation for extending the shelf life of bread.

## 2. Materials and Methods

### 2.1. Materials

*Lactobacillus rhamnosus* HCUL 1.1901–1912 (isolated from Yunnan-fermented tofu, identified as *Lactobacillus rhamnosus*, and preserved in the Laboratory of the College of Food Engineering), *Lactococcus lactis* subsp. *lactis* (strain: 1.2472), and *Streptococcus thermophilus* (strain: 1.2718) were purchased from China General Microbiological Culture Collection Center (CGMCC); Japonica rice (protein: 12%, fat 1%, carbohydrate: 28%, moisture: 14%) was purchased from Heilongjiang Yifeng Commodity Trading Co., Ltd. (Yifeng, Harbin, China).; wheat flour (protein: 18%, fat: 3%, carbohydrate: 25%) was purchased from Qingdao Bailemai Food Co., Ltd. (Bailewei, Qingdao, China). Milk powder (food grade) was purchased from Bright Dairy Co., Ltd. (Guangming, Harbin, China); butter (food grade) was purchased Shanghai DeYang Co., Ltd. (Deyang, Shanghai, China); angel yeast, sugar, and salt (food grade) were all purchased from a local market; MRS broth medium, sodium hydroxide, hydrogen chloride, ethanol, and sodium chloride were all analytically pure reagents.

### 2.2. Preparation of Fermented Rice Flour and Rice Starch

#### 2.2.1. Microorganisms and Culture

*Lactobacillus rhamnosus* (strain HCUL 1.1901–1912), *Lactococcus lactis* subsp. *lactis* (strain 1.2472), and *Streptococcus thermophilus* (strain 1.2718) were stored at −80 °C in 25% glycerol. Prior to use, cultures were aseptically thawed and activated for 24 h in sterile MRS broth at 37 °C till the viable count reached about 108 cfu/mL, and then these were used in the following analyses [21,22].

#### 2.2.2. Preparation of Fermented Rice Flour and Rice Starch

A conical flask was charged with a mixture of *Streptococcus thermophilus* fermentation suspension (5%), *Lactococcus lactis* subsp. *lactis* fermentation suspension, and thermostable *Lactobacillus rhamnosus* fermentation suspensions, all prepared using distilled water as the baseline. The japonica rice to distilled water ratio was 1:4. Specifically, the flask was then incubated at 37 °C for 1 d. The prepared fermented rice broth of *Lactococcus lactis* subsp. *lactis* rice flour (LRF) was homogenized, centrifuged at 4000 r/min for 15 min, and the supernatant was discarded. The obtained precipitate was dried at 45 °C in a hot-air dryer for 24 h, and then crushed with a pulverizer and sieved using an 80-mesh screen to obtain the LRF [23]. Thermostable *Lactobacillus rhamnosus*-fermented rice flour (TRF) and *Streptococcus thermophilus*-fermented rice flour (SRF) were prepared using the same method.

Rice starch (RS) was prepared as follows: Rice flour was added into 0.2 g/100 mL NaOH solution at a solid–liquid ratio of 1:3 (g/mL). The mixture was extracted for 3 h and centrifuged at 3000 r/min for 10 min. The supernatant was discarded and the upper layer of the brownish material in the sediment zone was removed. The residue was washed with water and centrifuged four times until the starch paste turned white. The slurry was adjusted to pH 7.0 with 1 mol/L HCl, centrifuged, dried at 30 °C, and sieved through an 80-mesh screen, and thus rice starch was obtained [24]. *Lactococcus lactis* subsp. *lactis* fermented rice starch (LRS), *Streptococcus thermophilus* fermented rice starch (SRS), and thermostable *Lactobacillus rhamnosus* fermented rice starch (TRS) were prepared using the same method. Rice flour (RF) was used as a control and did not require further treatment.

### 2.3. Production Process of Fermented Rice Bread

The bread ingredients (Table 1) were mixed in a laboratory-scale mixer (Kitchenaid, Artisan Series, Greenville, OH, USA) until a homogeneous fluid dough consistency was achieved in 5 min. Each dough portion weighed 50 g and was subjected to a quality fermentation for 2 h at 37 °C and 75% relative humidity. The fermented dough was baked at 170 °C for 15 min (Bosch HGD52D120T, Istanbul, Turkey). After baking, the bread was cooled for 1 h and then packed in polyethylene bags.

### 2.4. Determination of Amylose

To analyze the content of the three types of starches, the methods described by Li et al. [25] were used, following the manufacturer’s instructions (Solarbio Science and Technology, Beijing, China).

### 2.5. Determination of RVA Viscosity Properties

The viscosity of the samples was determined using a rapid viscosity analyzer (RVA) according to the AACC Approved Method 61–02 (2000). Each RVA sample measurement container comprised 3 g of the sample and 25 mL of distilled water. The sample measurement container was placed in an RVA testing instrument equipped with a paddle, and stirred at a constant speed of 160 rpm. The temperature control software was activated, raising the starch slurry temperature to 50 °C to start the timing. The temperature was held at 50 °C for 1 min, then increased to 95 °C (reached at 4.75 min), followed by a 2.5 min holding period at 95 °C (until 7.25 min). Subsequently, the temperature was decreased to 50 °C (by 11 min) and maintained until 16 min [26,27,28]. The RVA provided the following parameters: peak viscosity (PV), trough (T), breakdown (BD), final viscosity (FV), and setback (SB).

### 2.6. Fourier Transform Infrared (FT-IR) Spectroscopy

A mixture of the three different rice starches (6.0 mg) and 200 mg of KBr (spectroscopic grade) was ground into a fine powder and pressed into KBr pellets. Infrared spectra were recorded using a FT-IR spectrometer (Spectrum100, Perkin-Elmer, Waltham, MA, USA) with a resolution of 4.0 cm^−1^ and scanning range of 4000–400 cm^−1^. Absorbance ratios of 1047/1020 cm^−1^ and 995/1020 cm^−1^ were calculated from the deconvoluted spectra [29,30].

### 2.7. Effect of Fermentation on Molecular Weight of Rice Starch

The molecular weight distribution of the three rice starches was measured as per the method of [31]. Samples (20 mg) were dissolved in 10 mL 90% DMSO Solution (*v*/*v*), gelatinized in a boiling water bath for 1 h, and stirred at 30 °C for 48 h. Thereafter, the samples were passed through a 0.45 μm filter and the filtrate (200 μL) was injected into a high-performance liquid exclusion chromatography system, equipped with a multi-angle laser light scattering (MALLS) detector (Wyatt Technologies, Santa Barbara, CA, USA), a refractive index (RI) detector (Wyatt Technologies, Santa Barbara, CA, USA), and Ultra hydrogel TMLinear 300 mm × 7.8 mmid × 2 (×2 means two columns in series). The mobile phase was a 0.1 mol/L NaNO_3_ solution containing 0.02% NaN_3_. The temperature of the column was set as 30 °C.

### 2.8. X-ray Diffraction

Diffraction patterns of the samples were acquired using XRD (D/Max2550VB+PC Rigaku Corp., Rigaku, Japan) at 30 kV. The samples were scanned at 10 mA in a range of 4–40° (2θ) using Cu-Kα radiation. The relative crystallinity of the starch granules was calculated as the ratio of the sample peak area to the total diffractogram area using JADE software (version 5.0; Materials Data Inc., Livermore, CA, USA) [32,33].

### 2.9. Differential Scanning Calorimetry (DSC)

Differential scanning calorimetry was used to measure the thermal properties of rice starch including the gelatinization onset temperature (To), gelatinization peak temperature (Tp), gelatinization completion temperature (Tc), and gelatinization enthalpy (ΔHgel). A measure of 3 mg rice starch was mixed with 7 μL distilled water in an aluminum pan, and was sealed hermetically and equilibrated overnight at 4 °C. The heating range was 20−100 °C at a rate of 10 °C/min. An empty pan was used as the reference. All samples were analyzed in triplicate.

Completely gelatinized rice starch was stored at 4 °C for 7 days, and the To, Tp, Tc, and staling enthalpy (ΔHret) of the crystals formed by staling were measured. The degree of staling was calculated using the following equation:

$$RD\%=\frac{\Delta Hret}{\Delta Hgel}\times 100$$

In the formula: ΔHret is the staling enthalpy/(J/g); ΔHgel is the gelatinization enthalpy (J/g).

### 2.10. Low Field NMR

A low-field (21 MHz) 1H nuclear magic resonance spectrometer (model: NMI20 Magnetic Resonance Installing Analyzer: Newmai Technology, Suzhou, China) operating at 32.0 ± 0.1 °C was used to study the range of proton molecular mobility by measuring the transverse (T_2_) relaxation time and the fraction of signal amplitude of protons in each part of T_2_. Three grams of breadcrumb (15 mm high, extracted from loaf center) were placed in a 40 mm NMR tube, which was then sealed with Parafilm^®^, to prevent moisture loss during the NMR experiment. The parameters used in the CPMG sequence: the proton resonance frequency was 22.6 MHz, the number of echoes C0 = 3000, the number of repeated scans NS = 8, and the half-echo time TE = 0.3 ms. Using CONTIN software 20 to call CPMG sequence inversion, each peak time constant T_2i_ (peak top time) and its area fraction A2i were recorded for subsequent analysis [34].

### 2.11. Texture (TPA) Determination

The hardness and elasticity of the breadcrumbs were measured using a TA.new+plus texture analyzer (TA Instrument Company, Shanghai, China). The central part of the bread was cut into a cube (2 × 2 × 2 cm^3^) and compressed (trigger force = 5 g) to 50% deformation with a cylindrical probe (P/35 DiaCylinder Aluminum) and the following parameters: pre-test speed (2 mm/s), test speed (1 mm/s), and post-test speed (2 mm/s). Hardness (height of the first compression peak) and elasticity (ratio of second/first compression peaks) were determined [35].

### 2.12. Data Analysis

All tests and treatments were performed in triplicate unless otherwise stated. SPSS 25.0 was used for part of the data analysis. Data were expressed as mean ± standard deviation, and one-way analysis of variance was applied to detect treatment effects. The significance level was set at *p* ≤ 0.05. Duncan’s test was used to compare treatments with the control at a 5% significance level. Graph analyses were performed using Origin 9.0 software.

## 3. Results and Discussion

### 3.1. Effects of Fermentation on Physicochemical Properties of Rice Flour

#### 3.1.1. Amylose Content

**Figure 1:**
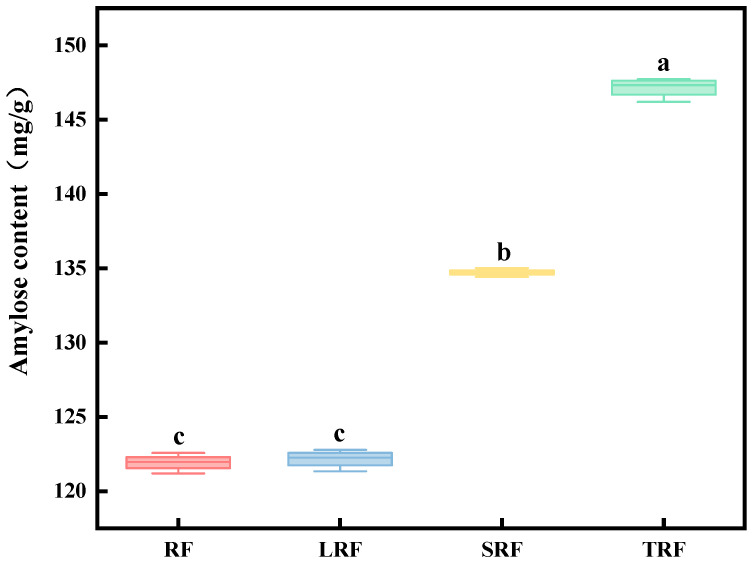
Amylose content in rice flour fermented using different strains (the values are expressed as mean ± SD (*n* = 3)). Values with the same letter at each addition are not significantly different at *p* < 0.05.

The amylose content of rice flour was determined using an amylose reagent kit. As shown in Figure 1, the amylose content increased in both SRF and TRF after fermentation. Among them, the highest amylose content in TRF was 147.50 mg/g, followed by SRF at 135.01 mg/g, while LRF showed minimal changes in amylose content. This was primarily due to the organic acids produced during fermentation, which initially act on the outer branches of the amylopectin molecules, leading to their degradation and the formation of amylopectin or amylose. This process resulted in increased amylose content in the fermented rice flour. Additionally, research has indicated that although starch-metabolizing glycoside hydrolase genes are widely present in *Lactococcus lactis* subsp, these strains lack the ability to utilize starch [36]. This finding is consistent with similar studies by Lu et al. [37], suggesting that branched starch undergoes cleavage or debranching during fermentation, resulting in the formation of amylose.

#### 3.1.2. RVA Gelatinization Characteristics

**Figure 2:**
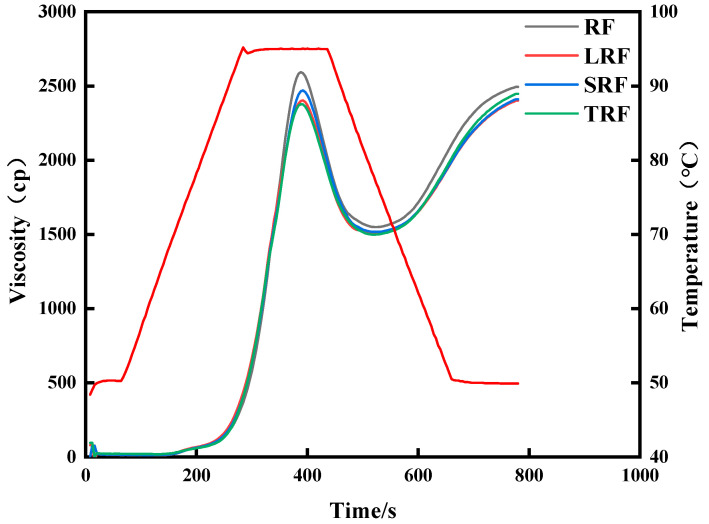
Changes in RVA gelatinization characteristics of rice flour fermented using different strains.

**Figure 3:**
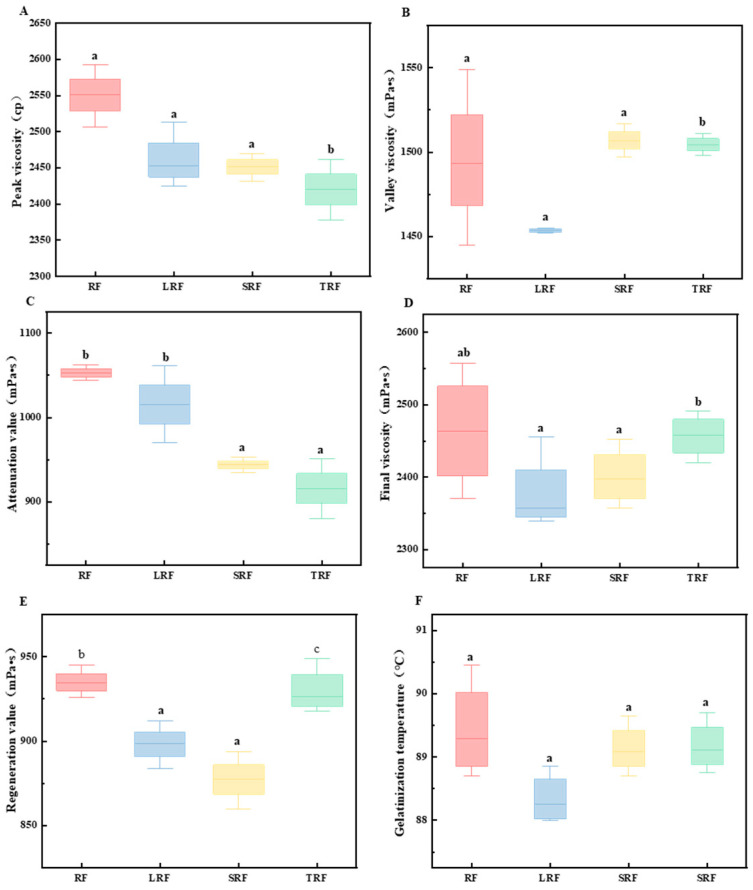
Gelatinization characteristic parameters of rice flour fermented using different strains ((**A**) peak viscosity; (**B**) valley viscosity; (**C**) attenuation value; (**D**) final viscosity; (**E**) regeneration value; (**F**) gelatinization temperature) (the values are expressed as mean ± SD (*n* = 3)). Values with the same letter at each addition are not significantly different at *p* < 0.05.

**Figure 4:**
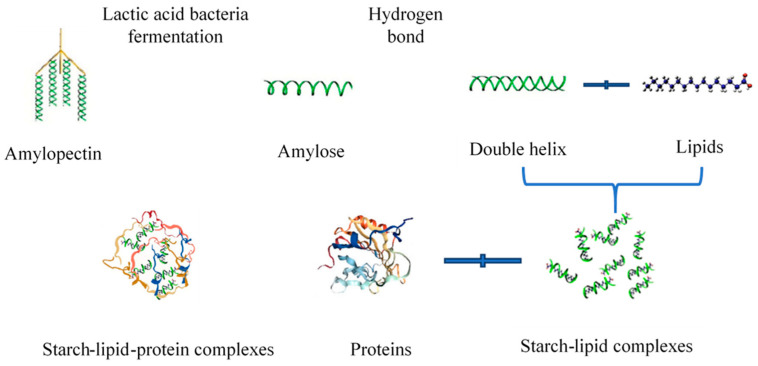
Schematic diagram of spiral inclusion complex of starch–fat and complex of starch–fat–protein.

Figure 2 and Figure 3 illustrate the changes in the RVA gelatinization characteristics of rice flour fermented with different strains. As shown in Figure 2, the strains affected the gelatinization process of rice starch, with fermentation having the greatest influence on the peak and trough viscosities of the SRF and TRF rice pastes. Figure 3 shows that the peak viscosity (Figure 3A), breakdown value (Figure 3B), final viscosity (Figure 3C), setback value (Figure 3D), and gelatinization temperature (Figure 3F) of the rice flours fermented by the three strains decreased. Meanwhile, the trough viscosity increased for both SRF and TRF. In the fermentation process, a significant amount of acid is produced, and among them, lactic acid can accelerate the dissolution of proteins due to its α-hydroxy structure. Lactic acid can form hydrogen bonds with functional groups on peptide chains [38]. This facilitates the dissolution of proteins. The reduction in rice protein content during lactic acid bacterial fermentation is primarily a result of the action of lactic acid. Therefore, during the initial gelatinization stage, proteins in rice flour primarily exist in the form of protein bodies. The swelling of starch granules during the heating process was inhibited, and fermentation led to the partial decomposition and dissolution of some proteins. This reduces the inhibitory effect of certain proteins on starch gelatinization, accelerates the rate of water absorption and swelling during starch gelatinization, enhances starch hydration, accelerates starch gelatinization, and lowers the gelatinization temperature. Yang et al. [39] found that fermentation decreased the peak viscosity of rice flour. This can be attributed to several factors during the fermentation. On the one hand, lactic acid bacteria hydrolyzed the short chains of branched starch, resulting in a reduction in the short/long chain ratio of amylopectin. This led to a decrease in the average chain length and degree of polymerization of amylopectin, causing the starch molecules to become smaller and reducing spatial hindrance. Subsequent molecular weight studies confirmed this observation. On the other hand, non-starch components such as proteins, lipids, and β-glucans also influenced the gelatinization characteristics of rice flour [40]. Various organic acids and enzymes produced by microbial activity during fermentation break down the proteins. During the gelatinization process, the proteins that encapsulated starch (Figure 4) leached out, releasing starch. Under the influence of shear forces, starch is more likely to form orderly arrangements, leading to a reduced shear resistance [41].

The breakdown values of the rice flour fermented by the three strains exhibited varying degrees of reduction, with TRF showing the greatest decrease. The breakdown value reflected the stability of the starch granule structure during heating. The decrease in breakdown value indicated an enhanced ability of fermented rice starch to maintain granule structure integrity during heating [42]. The long-chain portion of amylopectin was negatively correlated with its breakdown value during gelatinization, whereas the short-chain portion was positively correlated with the breakdown value [43]. Longer chains may provide stronger interlinking to maintain the rigidity of the swollen starch granules during heating and stirring, making them less susceptible to breakage. The change in the length of amylopectin chains after fermentation may be a significant factor contributing to the reduction in starch breakdown.

The final viscosity is an assessment of the stability of the cold paste under low-shear conditions, and a smaller value indicates poorer shear resistance stability of starch. The final viscosity of rice flour fermented by the three strains decreased, possibly because of a reduction in the molecular weight of starch. Moreover, TRF > SRF > LRF followed the same pattern as the amylose content, as amylose was prone to self-aggregation and was a major contributor to short-term staling. This suggests that amylose content likely has a significant impact on the final viscosity.

The control rice flour paste exhibited a setback value of 935 mPa·s. The lowest setback value was observed for SRF at 877 mPa·s, followed by LRF at 898 mPa·s, and TRF had a setback value of 923 mPa·s. The setback values of the rice flour fermented by the three strains decreased, indicating that fermentation altered the chemical composition and starch structure of the rice flour, affecting its setback characteristics and reducing its short-term retrogradation ability. Li et al. [44] found, in their study, that all strains, based on fermentation methods, could lower the final viscosity and short-term setback of rice starch. This could be attributed to the production of starch enzymes by the fermentation strains, causing disruption of the granular structure in the amorphous region of the starch particles. This enzymatic action led to the hydrolysis of amylose into smaller molecular states, resulting in rapid dissociation that prevented the formation of stable double-helical structures, ultimately reducing the setback value [45]. The final viscosity is an evaluation of the stability of the cold paste under low shear: the smaller the value, the worse the shear stability of the starch. The final viscosity of the fermented rice flour decreased.

The smallest difference between the peak and trough viscosities was observed for TRF, indicating that it had the least shear thinning, which resulted from the combination of starch granule swelling and collapse upon high-temperature water absorption and shear forces. This suggests that the TRF starch paste exhibits strong resistance to shear forces under high-temperature conditions. This is likely related to the content and distribution of amylopectin within the starch structure.

In summary, lactic acid bacteria play a pivotal role in the generation of both acids and enzymes during rice fermentation. These factors collectively alter the properties of starch granules. Concurrently, the molecular structure of starch undergoes modifications involving the hydrolysis of amylopectin and amylose in rice. This results in a reduction in the molecular weight of amylose and the degradation of protein and fat components. Consequently, these changes influence the gelatinization characteristics of fermented rice flour, ultimately enhancing its anti-aging properties.

### 3.2. Effects of Fermentation on Physicochemical Properties of Rice Starch

#### 3.2.1. Molecular Weight of Starch

Table 2 shows the molecular weight and distribution of rice starch fermented by different lactic acid bacteria. The molecular weight of rice starch is divided into two regions: region I (mainly amylopectin) and region II (mainly amylose with a small amount of amylopectin). The number–average molecular weight (Mn) and weight–average molecular weight (Mw) of SRS and TRS in regions I and II were lower than those of rice starch, indicating that fermentation caused the hydrolysis of amylopectin and amylose in regions I and II and the formation of small molecular substances that could be utilized by microorganisms. Meanwhile, the molecular weight and retro gradation value decreased. The number–average molecular weight of LRS in region I increased by 7608 g/mol^−1^, the weight–average molecular weight decreased by 1898 g/mol^−1^, and the number–average molecular weight and weight–average molecular weight of region II decreased by 176 g/mol^−1^ and 518 g/mol^−1^, respectively. This is because fermentation can hydrolyze short amylopectin in region I and increase the proportion of long amylopectin, thereby purifying the amylopectin. At the same time fermentation made the amylose in zone II hydrolyzed, and the retro gradation value of starch decreased. After fermentation, the molecular weight distribution (Mw/Mn) of the zone I starch was slightly lower than that of rice starch, indicating that the molecular chain length of zone I rice starch was altered by fermentation. The molecular weight distribution of rice starch in zone II narrowed, indicating that fermentation made the amylose component in zone II singular and played a role in purifying the starch. At the same time, the retro gradation value of starch decreases, playing an anti-staling role. It has been shown that fermentation reduces the molecular weight of millet starch, resulting in the partial hydrolysis of amylopectin in region I and a decrease in amylose content in region II [45].

#### 3.2.2. Fourier Transform Infrared Spectroscopy

Fourier transform infrared spectroscopy is widely used because of its sensitivity to changes in starch molecular conformation, enabling the analysis of the molecular structure of starch [46]. Infrared spectroscopy was employed to study the alterations in the functional groups and chemical bonds of rice starch molecules in different fermentation strains, as shown in Table 3. The absorption peaks of C-O were observed in the range of 1000–1800 cm^−1^, -CH absorption peaks appeared in the range of 2800–3100 cm^−1^, and -OH absorption peaks were seen in the range of 3200–3500 cm^−1^.

**Figure 5:**
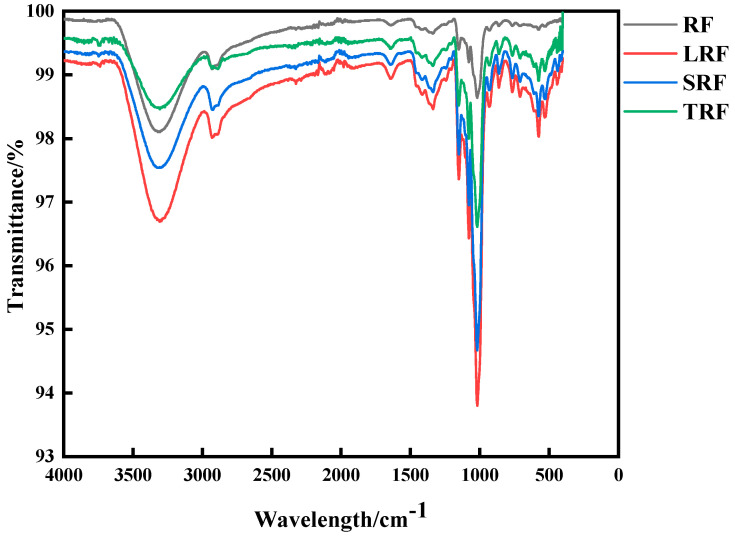
Fourier transform infrared spectra of rice starch fermented using different strains.

The infrared absorption spectra of rice starch fermented by the three strains and the unfermented rice starch showed no significant differences in peak shape at Figure 5. No new absorption peaks were observed; however, to some extent, the absorption peak intensities changed. The order of the absorption peak intensities was as follows: LRF > SRF > TRF > RF. This indicated that no new chemical bonds or functional groups were formed during fermentation.

Through infrared structural analysis, the deconvolution of the absorption peaks was performed to analyze the short-range ordered structure of the starch molecules. Park et al. [47] found that the infrared absorption at 1022 cm^−1^ represented the structural characteristics of the amorphous region of starch, whereas the infrared absorption at 1047 cm^−1^ represented the structural features of the crystalline region of starch molecules. The absorbance ratio represented the degree of order of the starch double helix arrangement [48]. The transmittance ratios at 1047/1022 cm^−1^ and 995/1022 cm^−1^ represent changes in the internal order (DO) and double helix content (DD) of the starch molecules, respectively. A greater transmittance at 995 and 1047 cm^−1^ or a lower transmittance at 1022 cm^−1^ indicated an increase in the crystallinity of the starch structure [49].

**Figure 6:**
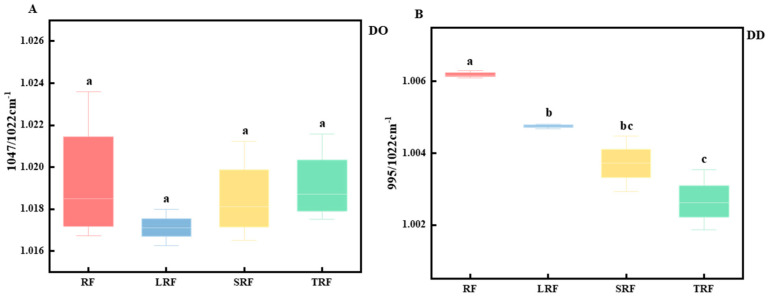
Analysis of degree of order (DO) (**A**) and double helicity (DD) (**B**) values of rice starch fermented using different strains (the values are expressed as mean ± SD (*n* = 3)). Values with the same letter at each addition are not significantly different at *p* < 0.05.

From Figure 6A,B, it can be observed that, compared to RF, the DO and DD values of fermented rice starch were reduced. LRF exhibited the greatest reduction in DO, followed by SRF and TRF. Among them, TRF showed the greatest reduction in the DD value, followed by SRF and LRF. The changes in the DO and DD values of fermented rice starch were mainly due to alterations in the ordered structure of some starch molecules after fermentation. Fermentation modifies the chain distribution of branched starch, thereby reducing its short-range order [50,51]. Yang et al. [51] obtained similar results in their experiments on lactic acid bacterial fermentation of corn starch, confirming the degradation of branched starch through fermentation.

#### 3.2.3. X-ray Diffraction

Starch is a highly ordered macromolecule comprising crystalline and amorphous regions. X-ray diffraction (XRD) patterns were used to analyze the long-range order of starch. The crystalline structure represents a long-range order and is characterized by distinct diffraction peaks. In the crystalline region, the grain size is large, and the crystal form is well ordered. In contrast, the amorphous region (also known as the non-crystalline region) is characterized by short-range order and long-range disorder with obvious diffuse scattering features [52].

**Figure 7:**
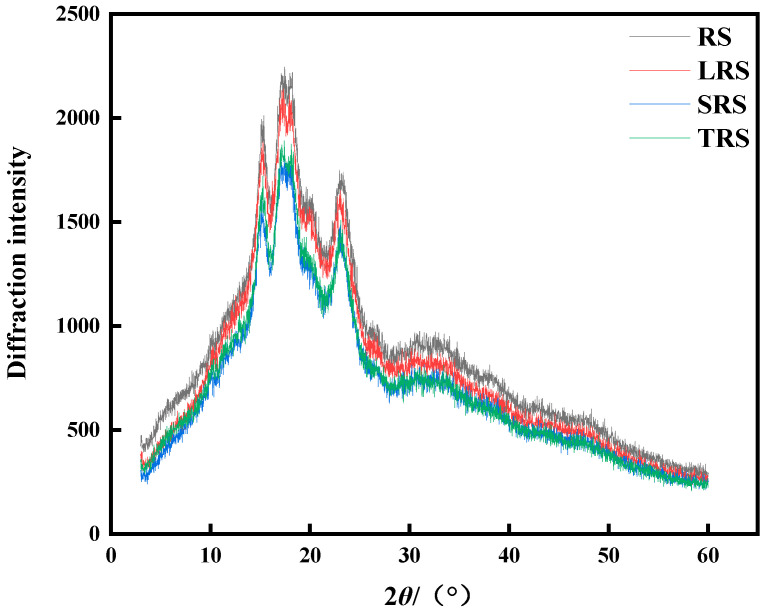
X-ray diffraction pattern of fermented rice starch.

As indicated in Table 4, the diffraction angles of fermented rice starch remained unchanged after fermentation. Distinct diffraction peaks appeared at 15°, 17°, 18°, and 23° with little difference in the crystal plane spacing. As shown in Figure 7, the peak intensities of fermented rice starch were significantly reduced. No new peaks emerged, indicating that the starch remained A-type crystalline after fermentation and that fermentation did not alter the crystalline structure of rice starch. The crystallinity of fermented rice starch was lower than that of unfermented rice starch. The decrease in crystallinity is beneficial for reducing starch staling because the crystalline region acts as a nucleus for staling. Through starch molecule movement, more starch molecules form regular arrangements through bonding near the crystalline region, enlarging the nucleus and contributing to staling effects [53]. The crystallinity of LRS was 11.64%, the lowest among the rice starch fermented by different strains, showing a reduction of 31.85% compared to unfermented rice starch. This suggests that fermentation has a limited effect on the long-range order of rice starch and possesses some anti-staling properties. Zhao et al. [45] found that naturally fermented millet starch retained an A-type crystal form, with a decrease of 1.56% in crystallinity compared to unfermented millet starch. The decrease in crystallinity of fermented rice starch was consistent with the experimental results of reduced DO and DD values.

#### 3.2.4. Differential Scanning Calorimetry (DSC)

Thermal properties can be employed to determine starch gelatinization, and in general, the thermal properties of starch are related to the content of linear starch in the crystalline region of starch granules, the distribution of branched starch chains, and the double helical structure of amylopectin chains [54].

**Figure 8:**
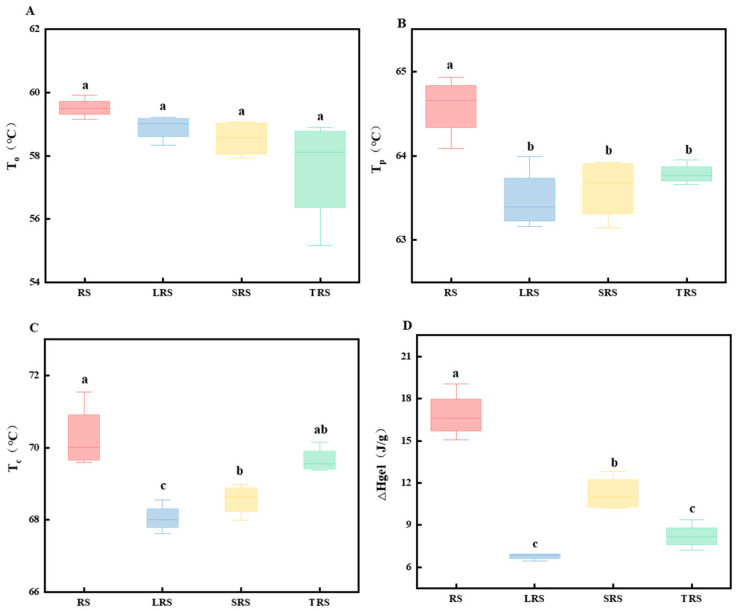
DSC thermal characteristic parameters of rice starch fermented by different strains ((**A**) onset temperature (To); (**B**) peak temperature (Tp); (**C**) end temperature (Tc); (**D**) enthalpy of gelatinization (ΔHgel)) (the values are expressed as mean ± SD (*n* = 3)). Values with the same letter at each addition are not significantly different at *p* < 0.05.

As shown in Figure 8, using thermal analysis methods, the gelatinization temperature of the starch samples in this experiment ranged from 57.57 to 58.99 °C, with a peak appearing between 63.48 and 64.58 °C. The onset temperature (To), peak temperature (Tp), end temperature (Tc), and enthalpy of gelatinization (△H) of the three types of fermented rice starch were all lower than that of RS. Among them, LRS had the smallest △H. In a previous RVA, LRS exhibited characteristics distinct from those of the other two types of fermented rice starch, as its linear starch content remained unchanged, and its crystallinity decreased significantly. This was likely the reason for the smallest △H value observed in LRS. The reduction in gelatinization temperature and enthalpy was attributed to the effect of microbial metabolism during fermentation. The acids and enzymes produced by microbial metabolism during the fermentation process act on the amorphous region of starch granules, leading to the disruption of the amorphous structure of starch. This enhances the water-holding capacity of starch, making it more susceptible to gelatinization. Furthermore, fermentation decreases the fat content of rice starch, thus weakening its ability to form complexes with starch [55,56]. Therefore, both the gelatinization temperature and enthalpy values of starch were reduced.

After gelatinization, rice starch undergoes transformation from a disordered to an ordered structure during storage, with rearrangements leading to recrystallization. The higher the staling enthalpy, the more pronounced is the recrystallization, indicating a more severe level of staling. Rice starch was assessed using DSC at different storage time intervals to analyze changes in its thermodynamic properties over the storage period.

**Figure 9:**
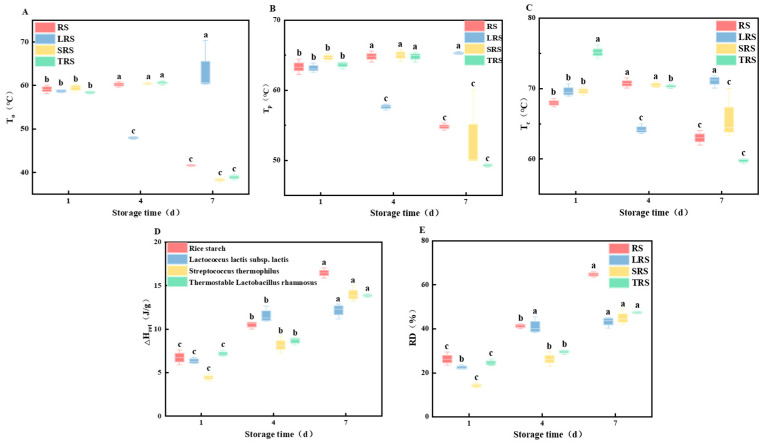
DSC parameters and staling degree of rice starch fermented by different strains at 4 °C at different times((**A**) onset temperature (To); (**B**) peak temperature (Tp); (**C**) end temperature (Tc); (**D**) enthalpy of gelatinization (ΔHgel) (**E**) degree of staling (RD)) (the values are expressed as mean ± SD (*n* = 3)). Values with the same letter at each addition are not significantly different at *p* < 0.05.

After staling, completely gelatinized fermented rice starch exhibited recrystallization between molecular chains, resulting in a single exothermic peak with higher symmetry during the heating process. As shown in Figure 9, the peak temperatures (To, Tp, and Tc) were higher than those of the corresponding gelatinization peaks, whereas the staling enthalpy was lower than the gelatinization enthalpy. With prolonged staling, the staling enthalpy gradually increased, indicating an increasing degree of starch staling. From Figure 9, it can be observed that the degree of staling of RS stored at 4 °C for 7 days was 64.68%, higher than that of fermented rice starch. Among the four types of starch, the anti-staling capability followed the order LRS > SRS > TRS > RS, with LRS having a staling degree of 43.22%.

### 3.3. Anti-Staling Properties of Fermented Rice Flour Bread

#### 3.3.1. Moisture Migration of Fermented Rice Flour Mass

**Figure 10:**
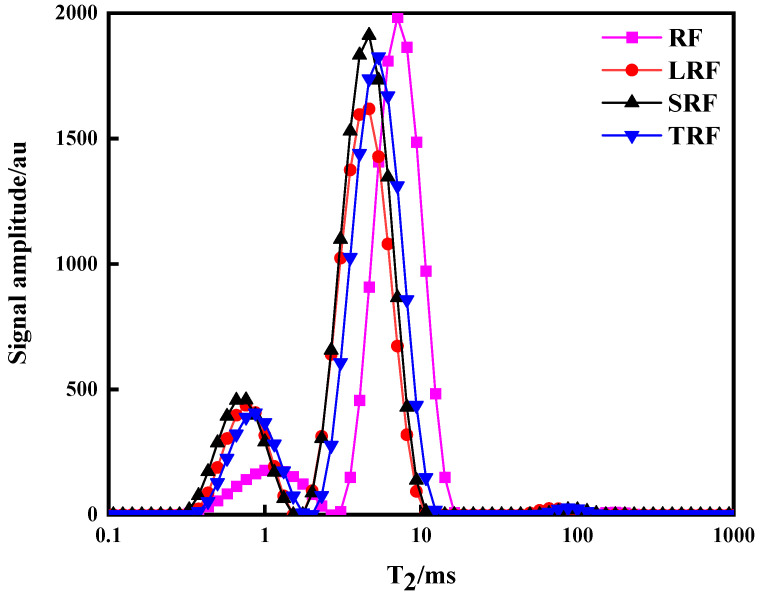
Inversion diagram of water transverse relaxation time T_2_ of rice dough made from rice flour fermented using different strains.

In Figure 10, each curve exhibits three peaks representing the three forms of moisture in the sample. T_21_ corresponds to bound water, primarily water tightly bound to starch or gluten proteins; T_22_ represents weakly bound water, flowing between bound water and free water, primarily associated with interactions among macromolecules such as proteins and starch; and T_23_ represents free water [57,58,59]. As shown in Figure 10, the amplitude of the T_21_ signal in the fermented rice flour dough was greater than that in RF, with SRF > LRF > TRF. The T_22_ peak was the main peak, accounting for approximately 80% of the total signal, indicating that in dough with well-formed gluten, the primary form of water was weakly bound. Differences were observed in the moisture forms and distributions of the four types of rice flour dough. The T_22_ peak times for the three fermented rice flour doughs were shorter than those for RF (7.10 ms), with TRF (5.34 ms) > LRF (4.64 ms) ≈ SRF (4.60 ms). From the above analysis, it can be concluded that dough made from RF had the highest water flowability, with water being loosely associated with other components, compared to dough made from fermented rice flour. Among the three types of fermented rice flour dough, LRF had the highest amplitude of the T_21_ signal and the shortest T_22_ peak time, indicating that the water flow ability was the weakest in the LRF dough, and that water was more tightly bound to other components.

The effects of different bacterial strains on the T_2_ and corresponding peak area A_2_ of the fermented rice flour dough are shown in Table 5. Longer transverse relaxation times indicate higher water mobility within the dough and less compact boundaries, whereas shorter transverse relaxation times imply tighter binding between water and substances. A_21_ represents water within starch granules or tightly bound to proteins, whereas A_22_ represents water outside the starch granules or within the protein network [60]. A_21_ in the fermented rice flour dough was consistently higher than that in the RF dough, whereas A_22_ was lower, indicating a tighter association between water and other components of the fermented rice flour dough, resulting in lower water mobility. The interaction between water and starch is pivotal in determining the fluidity of water in the dough, as proposed by Doona et al. [61]. In this analysis, it was observed that the post-fermentation rice flour had looser starch granules, reduced crystallinity and order, and increased amylose content. When kneaded into dough, it becomes more prone to binding and retaining water. However, it has been suggested that the binding of water with proteins in the dough is tighter than that with starch [62]. This could be attributed to the high protein and wet gluten contents, dense gluten network, and strong water-binding capacity of gluten, leading to reduced water mobility in the dough. In conclusion, the enhanced binding of fermented rice flour with water and the variations in moisture content among the three types of fermented rice flour dough were closely related to changes in protein and starch characteristics after fermentation.

#### 3.3.2. Texture Properties of Fermented Rice Flour Bread

Texture parameters of bread include hardness, elasticity, chewiness, adhesiveness, cohesiveness, and viscosity, which are used to evaluate bread quality [63]. In this study, texture analysis focused on the hardness, elasticity, and chewiness of the fermented rice bread. Hardness and chewiness are negatively correlated with bread quality, whereas elasticity is positively correlated with bread quality [64].

**Figure 11:**
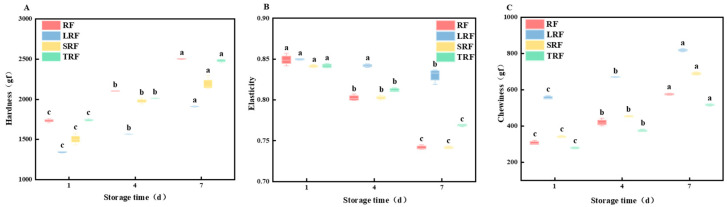
Changes in the texture properties of bread under different storage times ((**A**) hardness; (**B**) elasticity; (**C**) chewiness) (the values are expressed as mean ± SD (*n* = 3)). Values with the same letter at each addition are not significantly different at *p* < 0.05.

Hardness is the most important textural parameter of bread and serves as a crucial indicator to assess the level of bread staling. This represents the peak pressure during the first penetration of the sample [65]. Figure 11A shows that the hardness of RF rice flour bread was greater than that of fermented rice flour bread, and the hardness after staling was also higher. The hardness of bread increased with an increase in storage time. For example, the hardness value of fresh, unfermented rice bread was 1735.35 gf, which increased to 2504.75 gf after 7 days of storage at 4 °C, resulting in a hardness change of 1.441 times. Among the fermented rice flour breads, the change in hardness was 1.421 times, 1.426 times, and 1.428 times for LRF, SRF, and TRF, respectively, with LRF showing the smallest change, although the differences were not pronounced. The increase in bread hardness was primarily due to lower temperature and humidity during storage, which caused moisture migration within the bread, resulting in dryness. Additionally, starch molecules underwent retro gradation, transitioning from α-starch to β-starch during the storage process [66]. The relatively small change in the hardness for fermented rice bread was mainly attributed to the action of lipase produced by lactic acid bacteria during fermentation, which breaks down fats, leading to reduced fat content. Moreover, the breakdown of amylose into smaller molecules during fermentation leads to a decrease in amylose content, resulting in a reduction in the formation of amylose–fatty acid complexes, lower crystallinity, degradation–retrogradation values, and ultimately decreased hardness.

Elasticity reflects the extent of structural damage to the bread network during the first compression in TPA testing, and is an important indicator for evaluating bread staling [67]. As shown in Figure 11B, the elasticity of fresh RF rice flour bread was 0.85, which decreased to 0.74 after 7 d of storage, indicating a decrease of 12.94%. The elasticity decrease rate for LRF rice flour bread was 2.35%, for SRF rice flour bread it was 11.91%, and for TRF rice flour bread it was 8.33%. The results suggest that the elasticity decrease rates of fermented rice flour bread were lower than those of RF rice flour bread, with LRF exhibiting the smallest change in elasticity. This was mainly attributed to starch retrogradation and moisture loss during the bread process, leading to contraction of the bread gluten network structure and densification, thereby resulting in a decrease in elasticity [68]. This finding was consistent with that of Gerten et al. [20], who indicated that bread made with added lactic acid bacterial fermenting agents had higher elasticity during the shelf life than bread fermented only with yeast, suggesting that adding lactic acid bacterial fermenting agents helped increase bread elasticity and enhance the quality of the bread’s shelf life.

As shown in Figure 11C, the chewiness of bread increased with the number of storage days. For RF rice flour bread, the initial chewiness was 307.14 gf, which increased to 575.70 gf on the 7th day, indicating a change rate of 87.44%. The chewiness change rate for LRF rice flour bread was 47.07%, for SRF rice flour bread it was 102.19%, and for TRF rice flour bread it was 84.53%. During storage, bread tended to become drier, harder, and more difficult to chew, with a slightly sticky feeling, and significant breadcrumbs were observed during the compression process using a texture analyzer. However, among the fermented rice flour bread samples, the LRF rice flour bread showed the smallest change in chewiness, effectively improving this phenomenon.

## 4. Conclusions

In this study, lactic acid bacteria fermentation hydrolyzed short amylopectin, increased the proportion of longer amylopectin and amylose in rice flour, and degraded amylose. This resulted in a subsequent reduction in peak viscosity, setback viscosity, final viscosity, setback value, and gelatinization temperature of fermented starch. FT-IR and XRD analyses indicated a decrease in short- and long-range order for fermented rice flour, with LRS exhibiting the lowest crystallinity. DSC thermal analysis showed a general decrease in thermal parameters of rice starch, along with lowered staling degrees, with LRS having the smallest at 44.71%. Low-field NMR measurements revealed that water mobility in the fermented rice flour dough was limited, with tighter water interactions with other components, which was particularly pronounced in LRF. Texture analysis demonstrated that during storage, the hardness of non-fermented rice flour bread increased by a factor of 1.441, while fermented rice flour bread showed an increase by a factor of 1.421 for LRF, 1.426 for SRF, and 1.428 for TRF.

By investigating the physicochemical properties of fermented rice flour and rice starch, as well as the quality characteristics of dough and bread products, this study provides scientific evidence for the anti-staling properties of lactic acid bacteria-fermented rice flour and lays a theoretical foundation for extending the shelf life of rice bread. In this study, the anti-staling mechanism of rice bread prepared from fermented rice flour was analyzed only from the perspective of starch. However, further studies are required to investigate the mechanism of action of proteins and the interactions between proteins and starch in bread anti-staling.

## Figures and Tables

**Table 1:** Basic ingredients of bread.

Material	Addition Levels (*w*/*w*)
wheat flour	60%
rice flour	40%
sugar	18%
whole milk powder	6%
eggs	8%
water	50%
butter	8%
salt	0.6%
yeast	1.5%

**Table 2:** Molecular weight of fermented rice starch.

	Region	RF	LRF	SRF	TRF
Mn (g/mol^−1^)	I	50,836	58,444	44,032	38,811
Mw (g/mol^−1^)	I	111,560	109,662	90,334	72,034
Mn (g/mol^−1^)	II	2952	2776	2439	2091
Mw (g/mol^−1^)	II	5075	4557	4106	3403
Mw/Mn	I	2.19	1.88	2.05	1.86
	II	1.72	1.64	1.68	1.63

**Table 3:** Assignment analysis of each peak in Fourier infrared spectrum of rice flour fermented using different strains.

Peak Position/cm^−1^	Corresponding Group	Corresponding Group Position Range/cm^−1^	Peak Attribution
1010	V (C-O)	1800–1000	Symmetrical and asymmetric stretching vibration absorption peaks of C-O bond in glucose unit
1150			
1253			
1372			
1742			
2956	V (C-H)	3100–2800	C-H stretching vibration absorption peak
3296	V (O-H)	3500–3200	O-H stretching vibration absorption peaks in alcohol hydroxyl groups

**Table 4:** X-ray 2θ angle and peak width of rice starch fermented by different strains.

	Peak Number	RS	LRS	SRS	TRS
Diffraction angle 2*θ* (°)	1	15.269 ± 0.115 ^d^	15.162 ± 0.034 ^d^	15.263 ± 0.036 ^d^	15.247 ± 0.064 ^d^
	2	17.339 ± 0.097 ^c^	17.363 ± 0.075 ^c^	17.245 ± 0.085 ^c^	17.387 ± 0.106 ^c^
	3	18.130 ± 0.068 ^b^	18.105 ± 0.082 ^b^	18.056 ± 0.008 ^b^	18.204 ± 0.037 ^b^
	4	23.262 ± 0.062 ^a^	23.129 ± 0.111 ^a^	22.946 ± 0.060 ^a^	23.188 ± 0.067 ^a^
Crystal plane distance/nm	1	5.795 ± 0 ^d^	5.837 ± 0 ^d^	5.798 ± 0 ^d^	5.804 ± 0 ^d^
	2	5.109 ± 0 ^c^	5.101 ± 0 ^c^	5.136 ± 0 ^c^	5.094 ± 0 ^c^
	3	4.887 ± 0 ^b^	4.894 ± 0 ^b^	4.907 ± 0 ^b^	4.867 ± 0 ^b^
	4	3.819 ± 0 ^a^	3.841 ± 0 ^a^	3.871 ± ^a^	3.831 ± 0 ^a^
Crystallinity		17.08%	11.64%	13.31%	15.13%

Values are expressed as mean ± SD (*n* = 3). Values with the same letter at each addition are not significantly different at *p* < 0.05.

**Table 5:** Effects of different strains on moisture relaxation time T_2_ and corresponding peak area of fermented rice dough.

-	T_2_/ms			A/%		
-	T_21_/ms	T_22_/ms	T_23_/ms	A_21_/%	A_22_/%	A_23_/%
RF	0.26 ± 0.02 ^b^	2.66 ± 0 ^a^	114.98 ± 0 ^a^	11.12 ± 0.41 ^a^	88.61 ± 0.40 ^c^	0.26 ± 0.02 ^a^
LRF	0.33 ± 0.02 ^ab^	1.75 ± 0 ^a^	43.29 ± 0 ^c^	18.93 ± 0.05 ^c^	80.36 ± 0.08 ^a^	0.74 ± 0.21 ^c^
SRF	0.28 ± 0 ^ab^	1.75 ± 0 ^a^	52.25 ± 4.30 ^b^	18.74 ± 0.05 ^c^	80.65 ± 0.11 ^a^	0.60 ± 0.11 ^b^
TRF	0.31 ± 0.02 ^a^	2.01 ± 0 ^a^	47.61 ± 3.74 ^bc^	17.32 ± 0.12 ^b^	81.91 ± 0.07 ^b^	0.76 ± 0.50 ^c^

The values are expressed as mean ± SD (*n* = 3). Values with the same letter for each addition are not significantly different at *p* < 0.05.

## Data Availability

Data available on request from the authors.
